# Physicochemical and microbiological characteristics of urban aerosols in Krakow (Poland) and their potential health impact

**DOI:** 10.1007/s10653-021-00950-x

**Published:** 2021-04-29

**Authors:** Wanda Wilczyńska-Michalik, Anna Różańska, Małgorzata Bulanda, Agnieszka Chmielarczyk, Bartłomiej Pietras, Marek Michalik

**Affiliations:** 1 Institute of Geography, Pedagogical University in Kraków, ul. Podchorążych 2, Kraków, Poland; 2 Chair of Microbiology, Faculty of Medicine, Jagiellonian University Medical College, ul. Czysta 18, 31-121 Kraków, Poland; 3 Institute of Geological Sciences, Jagiellonian University, Ul. Gronostajowa 3a, 30-387 Kraków, Poland

**Keywords:** Urban aerosol, Atmospheric particulate matter, Size and morphology of particles, Chemical composition of particles, Microorganisms in aerosols, Aerosol health impact

## Abstract

Eight aerosol samples were collected in Krakow using a low-volume sampler in February and March 2019 during variable meteorological conditions and times of the day, to study their single particles’ properties (size, morphology and chemical composition analyzed using a scanning electron microscope fitted with an energy-dispersive spectrometer) and microbiological characteristics. The content of particles of different chemical compositions larger than 2.5 μm was low. Considering the number of the particles, submicron particles strongly dominated with a high content of ultrafine particles (nanoparticles). Tar ball-type particles were relatively common in the studied samples, while soot was the dominant component. Soot was present as small agglomerates composed of few particles, but also as bigger agglomerates. Metal-containing particles of various chemical characteristics were abundant, with transition metals commonly occurring in these particles. The physicochemical characteristics of aerosols indicate that despite a relatively low mass concentration, their adverse health impact could be very strong because of the high content of nanoparticles, the abundance of soot and other fuel combustion-related particles, and the high incidence of transition metal-rich particles. Microbiological analysis was based on cultures on both solid and liquid agar. The MALDI-TOF method was used for species identification—for bacteria and fungi. Twelve different species of bacteria were isolated from the collected samples of aerosols. The most frequently isolated species was Gram-positive sporulating *Bacillus licheniformis.* The isolated mold fungi were of the genus *Aspergillus*.

## Introduction

The percentage of urban populations is increasing rapidly. In 2018, the global urban population was 55.3% and it is expected to rise to 68.4% by 2050 (for more developed regions, 78.7% and 86.6%, and for less developed regions, 50.6 and 65.6%, respectively) (World Urbanization Prospects 2019). In Poland, the urban population constituted 60.05% of the total population in 2018, with a slight decrease observed between 2000 and 2018 (i.e., 61.88% in 2000 and 60.81% in 2010) (Population. Size and structure and vital statistics in Poland by territorial division …. 2019). High levels of air pollution are often noted in the urban environment, which results in a deterioration of environmental and health conditions (e.g., Kura et al. 2013). Urban areas play an important role in the emission of air pollutants (Jacobson 2012; Oke et al. 2017).

Lelieveld et al. (2015) estimated that 3.15 million premature deaths were related to outdoor PM2.5 globally in 2010. Taking into account the growth of urban populations and increasing air pollution concentrations, Lelieveld et al. (2015) estimated urban premature mortality to grow from 2.0 million in 2010 to 4.3 million by 2050. Global excess mortality related to ambient air pollution is estimated at ca. 8.8 million annually, with a reduction in life expectancy of 2.9 years (Lelieveld et al. 2020).

Epidemiological studies indicate an increase in morbidity and mortality due to air pollution (Ayres et al. 2008; Dellinger et al. 2000; Lodovici and Bigagli 2011; Valavanidis et al. 2008). However, less is known about what physical and chemical properties of particles negatively impact on health (Ayres et al. 2008). To study these relationships, a detailed physicochemical characterization of aerosol particles is needed. Airborne microorganisms influence cloud development, atmospheric chemistry but also the spread of numerous diseases (e.g., Deguillaume et al. 2008; Burrows et al. 2009), and identifying them is important in the evaluation of health impact.

The high concentration of air pollution in Krakow has been a problem of great concern since the 1960s. However, a decrease in the concentration of particulate matter (PM) and SO_2_ was noted during the last 30 years (Wilczyńska-Michalik et al. 2016). High concentration of PM exceeding the legal limits is often recorded in Krakow, mainly in cold seasons. NOx and benzo(a)pyrene are also important components of air pollution in the city. PM in Krakow is characterized by the domination of fine particles with a high abundance of soot. Single-particle studies indicate that PM is derived from various sources, both natural (e.g., soil erosion) and anthropogenic (e.g., industrial emission, emission related to the combustion of solid fuels in household heating systems, vehicular emission and others) (Wilczyńska-Michalik and Michalik 2015; Wilczyńska-Michalik et al. 2015a, 2016). Microbiological characteristics of atmospheric aerosols in Krakow are almost unknown.

The aim of this study is to present the identification of microbiological components of aerosols and physicochemical characteristics of non-biological particulate matter (i.e., particles’ size, morphology and chemical composition). Aerosols were collected in February and March 2019 in Krakow. Eight samples were collected during a two-week period, where the relatively short sampling period provided an opportunity to study the short-term variation in the samples’ composition related to changing meteorological conditions. The sampling period represented the cold season (with relatively high PM concentration) and partly coincided with the period of increased seasonal incidence (seasonal flu and other diseases) (Martinez et al. 2019; Moriyama et al. 2020; Meldunki epidemiologiczne 2020).

Individual particle analysis based on scanning electron microscopy and energy-dispersive spectrometry (SEM–EDS) was applied for determination of physicochemical properties of PM. Because of the analytical method used, only selected culturable microorganisms were determined. The fraction of airborne bacteria detected by culture methods is usually less than 10% (Burrows et al. 2009). Despite the limitations of the culture methods, the results are important in the studies of variation of the concentration of airborne bacteria with reference to seasons of the year, day and night, meteorological conditions (e.g., Bovallius et al. 1978; Fang et al. 2007; Dueker et al. 2017) or location in the town (Fang et al. 2007).

In this paper, we intend to point out those features of PM that are considered to be responsible for adverse effects on human health described in the literature, without detailed discussion on the interaction mechanism, or the possible environmental impacts.

## Methods

### Sampling

Samples of total suspended particulate matter (TSP) were collected on polycarbonate membranes (pore size 0.1 μm; 47 mm in diameter) using a Life 1 One (Mega System) sampler. Microbiological and physicochemical analyses were performed on fragments of each membrane (ca. 80% of the surface for microbiological analysis and 20% for physicochemical study). The sampling details are presented in Table 1.

**Table 1:** Sampling details

Date (dd/mm/yyyy)	Sampling time	Volume of air (l)	Localization
28/02/2019	9:00–16:30	3500	Campus
1/03/2019	8:00–15:45	3500	Campus
4/03/2019	8:30–16:20	3500	Campus
5/03/2019	8:35–16:40	3500	Campus
9/03/2019	15:25–24:20	4000	Friedleina
10/03/2019	12:00–19:40	4000	Friedleina
11/03/2019	15:10–23:35	4000	Friedleina
13/3/2019	8:30–17:50	5000	Campus

Campus—III Campus of the Jagiellonian University, 3a Gronostajowa Str.,

southern part of the city

Friedleina—22 Friedleina Str., northern part of the city

### Scanning electron microscopy with energy-dispersive spectrometry

Fragments of polycarbonate membranes were coated with carbon and gold. Carbon-coated fragments of polycarbonate membranes were used mainly for chemical analyses (using the EDS method) and imaging using backscattered and secondary electrons signals. Gold-coated fragments were used mainly for imaging using secondary electrons signal. A field emission scanning electron microscope (FESEM) (Hitachi S-4700) was used for imaging. Secondary electrons and backscattered electrons (with a YAG detector) imaging modes were applied. The chemical composition of the particles was determined using energy-dispersive spectrometry (EDS) Noran NSS system. Quantitative determination of the content of chemical elements in dust particles was based on the standardless method. All results were recalculated to 100% (without carbon content).

### Microbiological analysis

The polycarbonate membrane (47 mm in diameter), after the passage of 3500 or 4000 L of air, was placed on a sterile Petri dish, and a small piece with an area of 5 × 5 mm was cut out using sterile scissors for chemical testing. Then, the filter was divided into two equal parts.

One part of the filter was put on agar medium with the addition of blood (Columbia agar) for determination of the number of bacteria in atmospheric air, used in other Polish studies previously (Burkowska-But et al. 2014)] (Becton Dickinson GmbH, Germany) for qualitative culture. The medium was incubated at 36 ± 1 °C for 18 h. The next day, each morphologically different bacterial colony was isolated from the medium and inoculated into a new medium for later identification. Species identification was conducted using the MALDI-TOF method (Bruker Daltonics, Germany).

The second part of the filter was placed in liquid medium (tryptic soy broth [TSB]) and pre-incubated at 36 ± 1 °C for 18 h. After this, the portion of 100 µl was taken from the broth and spread on agar medium (tryptic soy agar [TSA]) and blood agar medium (Columbia agar) using the decimal serial dilution method. The colonies obtained by quantitative culture were counted, and the results were given as colony-forming unit per milliliter (CFU/ml). The colonies cultured and isolated in the quantitative part of the test were also identified using the MALDI-TOF method.

## Meteorological situation during sampling

The analyzed material contained a series of eight samples collected in the period from February 28 to March 13, 2019 (Table 1).

The temperature, humidity, wind speed and direction, insolation and cloud cover, together with the PM10 and PM2.5 concentrations measured at the State Environmental Monitoring Stations of the Provincial Inspectorate of Environmental Protection in Krakow are presented in Table 2. The relatively high wind speeds during the sampling period caused rather low concentrations of PM.

**Table 2:** Meteorological conditions and atmospheric dust concentration (PM10 and PM2.5) during sampling

Sample Date (dd/mm/yyyy)	Sampling time	Air temperature (^o^C)	Relative humidity (%)	Wind direction	Wind speed (m/s)	Cloudiness (oktas)	Atmospheric pressure (hPa)	Weather phenomena	Concentration of PM10* (μg/m^3^)	Concentration of PM2.5** (μg/m^3^)
28/02/2019	09:00	10.6	42.0	240	9.0	1	1007		59.4	31.3
	10:00	12.1	42.0	240	10.5	1	1006		55.3	24.0
	11:00	13.5	39.0	250	10.5	1	1006		54.7	19.0
	12:00	14.2	35.0	270	10.0	3	1005		59.7	21.0
	13:00	14.6	33.0	280	9.5	3	1005		66.4	21.7
	14:00	15.2	33.0	260	9.5	3	1005		53.6	20.0
	15:00	14.6	35.0	270	9.0	3	1004		51.4	18.3
	16:00	13.7	41.0	270	9.5	3	1004		44.7	15.3
	17:00	12.1	47.0	279	9.5	3	1004		39.7	16.0
1/03/2019	08:00	2.3	81.0	280	3.0	6	1009		65.1	49.0
	09:00	3.6	77.0	280	3.5	6	1009		63.6	43.3
	10:00	4.4	71.0	260	6.0	6	1009		65.3	40.7
	11:00	5.9	64.0	280	5.0	7	1008		58.6	36.3
	12:00	7.4	61.0	290	5.0	6	1008		56.3	36.3
	13:00	7.7	64.0	280	5.0	7	1008		56.1	34.7
	14:00	7.3	68.0	270	6.0	8	1007		58.3	38.3
	15:00	6.7	76.0	260	5.5	8	1007		68.0	46.0
	16:00	6	79.0	260	5.0	8	1007		77.3	54.7
4/03/2019	08:00	8.9	61.0	220	3.0	7	1004		29.9	16.0
	09:00	10.9	52.0	230	4.0	6	1003		35.6	13.0
	10:00	13.2	48.0	220	8.0	6	1003		32.1	12.7
	11:00	14.9	47.0	240	8.5	6	1002		27.0	9.3
	12:00	15	41.0	210	6.0	7	1001		24.3	8.7
	13:00	16.2	38.0	210	7.5	6	1000		20.3	7.3
	14:00	17.1	41.0	220	6.0	6	999		17.6	6.7
	15:00	16.6	44.0	230	6.5	7	998		19.9	6.3
	16:00	15.6	44.0	220	1.5	7	997		24.9	7.7
	17:00	14.8	49.0	variable	0.5	7	996		50.9	12.3
5/03/2019	08:00	5.3	59.0	230	9.5	6	1005		20.1	12.7
	09:00	7.1	56.0	240	12.5	5	1005		20.3	10.7
	10:00	8.1	53.0	240	13.0	3	1005	Wind gusts up to 18 m/s	20.1	13.0
	11:00	8.4	51.0	230	12.5	2	1005		19.7	9.3
	12:00	8.5	46.0	240	12.0	7	1006		21.0	7.3
	13:00	9.6	52.0	230	11.5	6	1006		18.4	7.7
	14:00	9.1	50.0	230	12.5	5	1006	Wind gusts up to 18 m/s	21.1	7.0
	15:00	8.7	81.0	240	11.5	7	1006		18.4	7.0
	16:00	5.8	76.0	260	5.5	4	1007	Shower rain	15.7	9.0
	17:00	6.1	69.0	240	4.5	6	1007		15.6	9.7
9/03/2019	15:00	8.3	67.0	230	7.5	7	1012		10.6	6.0
	16:00	8.9	66.0	230	8.5	7	1012		10.9	6.7
	17:00	8.9	69.0	220	6.0	7	1011		12.0	6.0
	18:00	8.1	69.0	220	6.5	7	1010		13.3	8.3
	19:00	8.6	77.0	220	8.0	8	1009	Shower rain	16.9	10.3
	20:00	8.1	83.0	230	7.5	8	1009	Shower rain	14.9	9.7
	21:00	7.3	81.0	230	6.5	8	1009	Shower rain	13.0	8.3
	22:00	7.2	77.0	230	4.5	7	1008		13.9	8.7
	23:00	7.2	90.0	220	4.0	7	1007		14.0	11.0
	00:00	5.7	79.0	210	4.5	8	1006	Shower rain	20.6	7.0
	01:00	7.7	81.0	230	9.5	7	1006		14.3	6.3
10/03/2019	12:00	9.2	49.0	240	9.5	7	1009		12.1	7.7
	13:00	9.4	74.0	240	8.5	7	1008		12.3	8.0
	14:00	6.9	85.0	220	8.0	8	1007	Light rain	13.7	7.3
	15:00	5.9	84.0	230	4.0	8	1006	Light rain	8.1	5.0
	16:00	6.2	84.0	variable	0.5	8	1004		8.7	6.7
	17:00	6.3	86.0	100	2.0	8	1002		11.9	9.3
	18:00	6	84.0	20	1.5	7	1000		13.9	14.7
	19:00	6.4	78.0	270	2.0	8	1000		15.9	11.3
	20:00	8.7	84.0	230	7.5	7	999		11.4	7.0
11/03/2019	15:00	7.7	42.0	230	6.5	5	1009		12.0	7.0
	16:00	6.2	53.0	250	7.0	7	1010		16.1	7.3
	17:00	3.1	83.0	230	11.5	8	1011	Shower rain with snow	15.1	5.3
	18:00	1.2	90.0	290	7.0	7	1012	Shower rain with snow	9.7	5.3
	19:00	1	91.0	250	4.0	5	1013		11.9	9.0
	20:00	0.6	93.0	200	3.0	4	1014		18.6	14.3
	21:00	0.5	91.0	230	3.0	2	1015		25.0	20.3
	22:00	0.7	89.0	220	3.5	7	1015		28.0	26.0
	23:00	1.1	93.0	260	5.0	7	1015	Light snow	27.2	26.7
	00:00	0.9	95.0	240	5.5	7	1015	Light shower snow	26.0	21.0
13/03/2019	08:00	1.3	65.0	variable	1.0	8	1010		51.6	19.3
	09:00	4.3	55.0	variable	1.5	8	1010		44.6	16.7
	10:00	7.2	50.0	210	3.5	8	1010		27.0	10.3
	11:00	7	57.0	220	7.0	8	1010		22.1	9.7
	12:00	6.4	62.0	220	3.5	8	1011		19.6	8.7
	13:00	6.3	61.0	230	5.0	8	1011		22.7	9.3
	14:00	7.6	56.0	230	6.5	6	1011		22.1	9.3
	15:00	8.3	56.0	240	7.0	5	1011		22.4	11.7
	16:00	8.1	59.0	210	6.0	6	1010		22.3	13.7
	17:00	7.3	66.0	210	4.0	6	1010		23.7	13.0

*Average from seven stations

**Average from three stations

The average wind speed during the sample collection varied from 4.5 to 9.7 m/s, while the average PM10 concentration during the sample collection was between 12.0 and 63.2 μg/m^3^. It is astonishing that during the collection of the samples on February 28, 2019, with a relatively strong wind (average value: 9.7 m/s) and low humidity, the concentration of PM10 was very high (average value: 53.9 μg/m^3^) (Table 2).

During the sampling period, in most cases, the weather was shaped by low-pressure systems, and among other dates, from March 9 to 11, 2019, the highest relative humidity values, favoring the formation of secondary aerosols (Zang et al. 2019), were recorded. The increase in relative humidity was probably related to the passage of an occluded atmospheric front system during the above-mentioned period. Significant activity of zonal circulation (western inflow) and, at the same time, the increase in pressure gradient connected with the passage of the front with wind speed and shower rain episodes, caused a significant dispersion and leaching of dust pollution from the atmosphere. As a result, relatively low dust concentration values were observed in the discussed period. However, the highest concentration of dust was observed on February 28, 2019, when Poland was under the influence of a trough associated with a quasi-stationary low over the northern part of Russia, and March 1, 2019, when the country was in a weak-gradient area of low pressure. Thus, on these dates, the north and northwest inflow of relatively dry and cold air masses dominated.

## Results and discussion

### Physicochemical characteristics of PM

#### Particle size

**Fig. 1:**
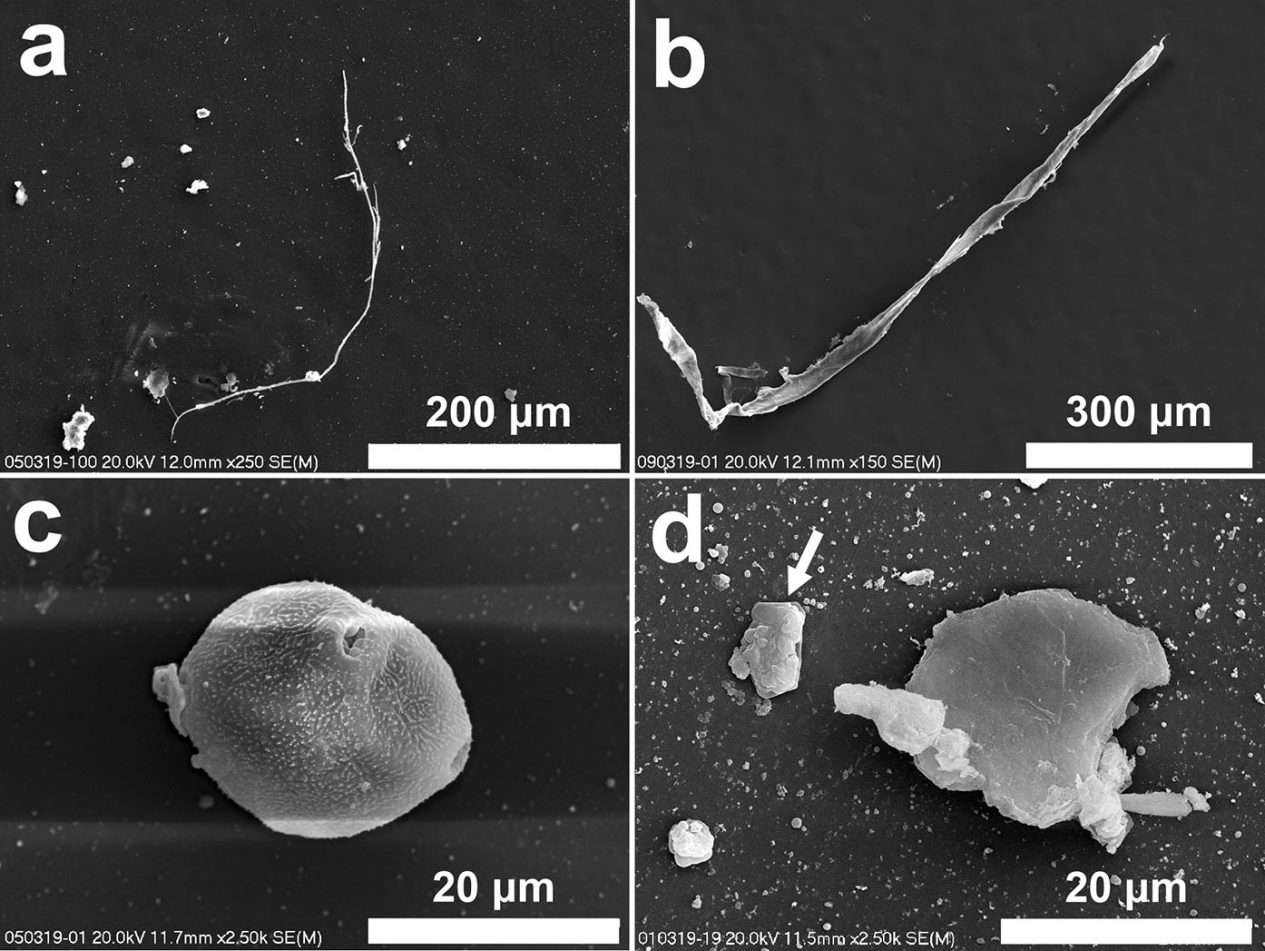
Coarse particles in atmospheric dust (SEM images). **a** and **b**. Fibrous plant debris. **c**. Pollen grain. **d**. Big aluminosilicate grain (containing Fe, Mg, Ti, Ca, K, Na); smaller particle (arrow) is composed of Cl and Na

**Fig. 2:**
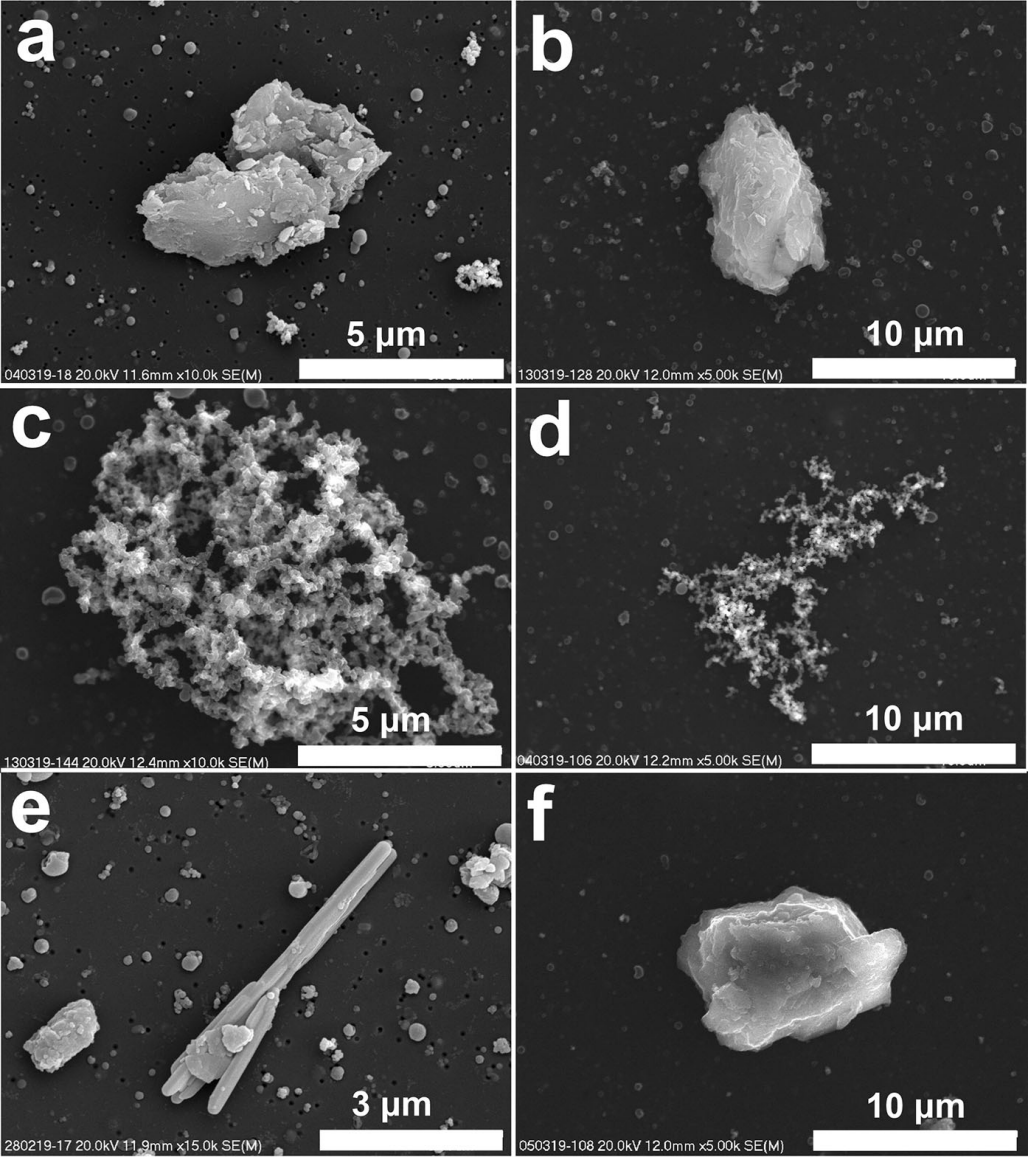
Particles of the size from 2.5 to 10 μm (SEM images). **a** and **b**. Aluminosilicate aggregates. **c** and **d**. Aggregates of soot particles. **e**. Elongated Ca sulfate grain. **f**. Ca and Mg carbonate

The method of sampling allowed the collection of the total suspended dust. Careful investigation of the samples indicated that only single particles larger than 10 μm are present. Plant debris fragments are usually present as fibrous material up to 0.5 mm long (Fig. 1a, 1b). Pollen grains are also often bigger than 10 μm (Fig. 1c). Aluminosilicate grains larger than 10 μm are scarce (Fig. 1d). The number of particles sized between 10 and 2.5 μm is very low. Their form of occurrence and the chemical composition of particles within this grain size are diversified. Aggregates of aluminosilicate particles (Fig. 2a, b) and aggregates of soot particles (Fig. 2c, 2d) dominate in this grain size category. Elongated Ca sulfate or Ca and Mg carbonate grains occur rarely (Fig. 2e, 2f). Single particles in aluminosilicates aggregates differ in chemical composition. Ca sulfates occur in some of the aggregates as grains or bind them.

**Fig. 3:**
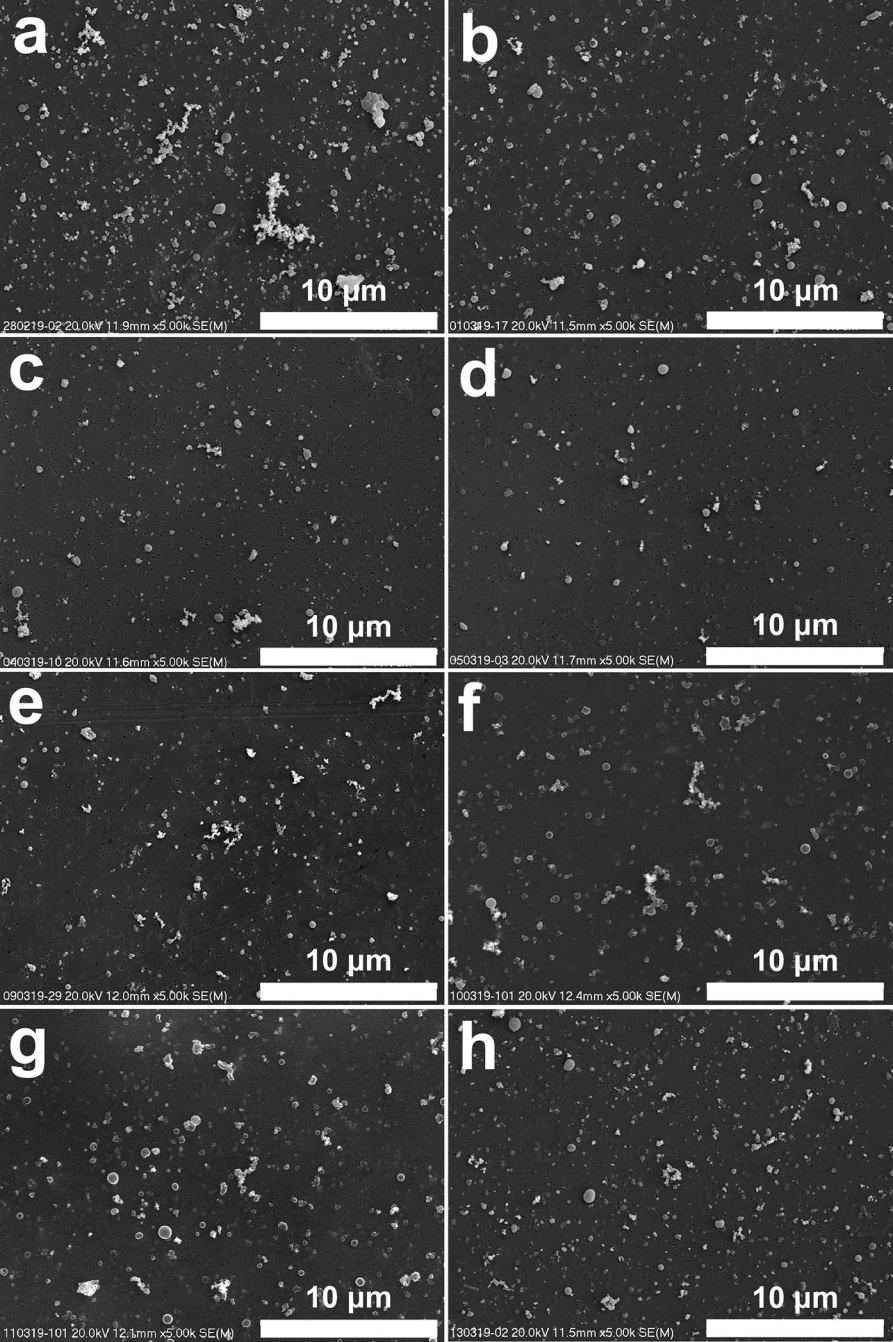
Atmospheric particulate matter on filter (SEM images, magnification 5 000 x). Dominance of particles below 1 μm. High content of soot and spherical tar ball-type particles. Very low content of particles above 1 μm. Images represent different samples: **a**—28/02/2019; **b**—01/03/2019; **c**—04/03/2019; **d**—05/03/2019; **e**—09/03/2019; **f**—10/03/2019; **g**—11/03/2019; **h**—13/03/2019

**Fig. 4:**
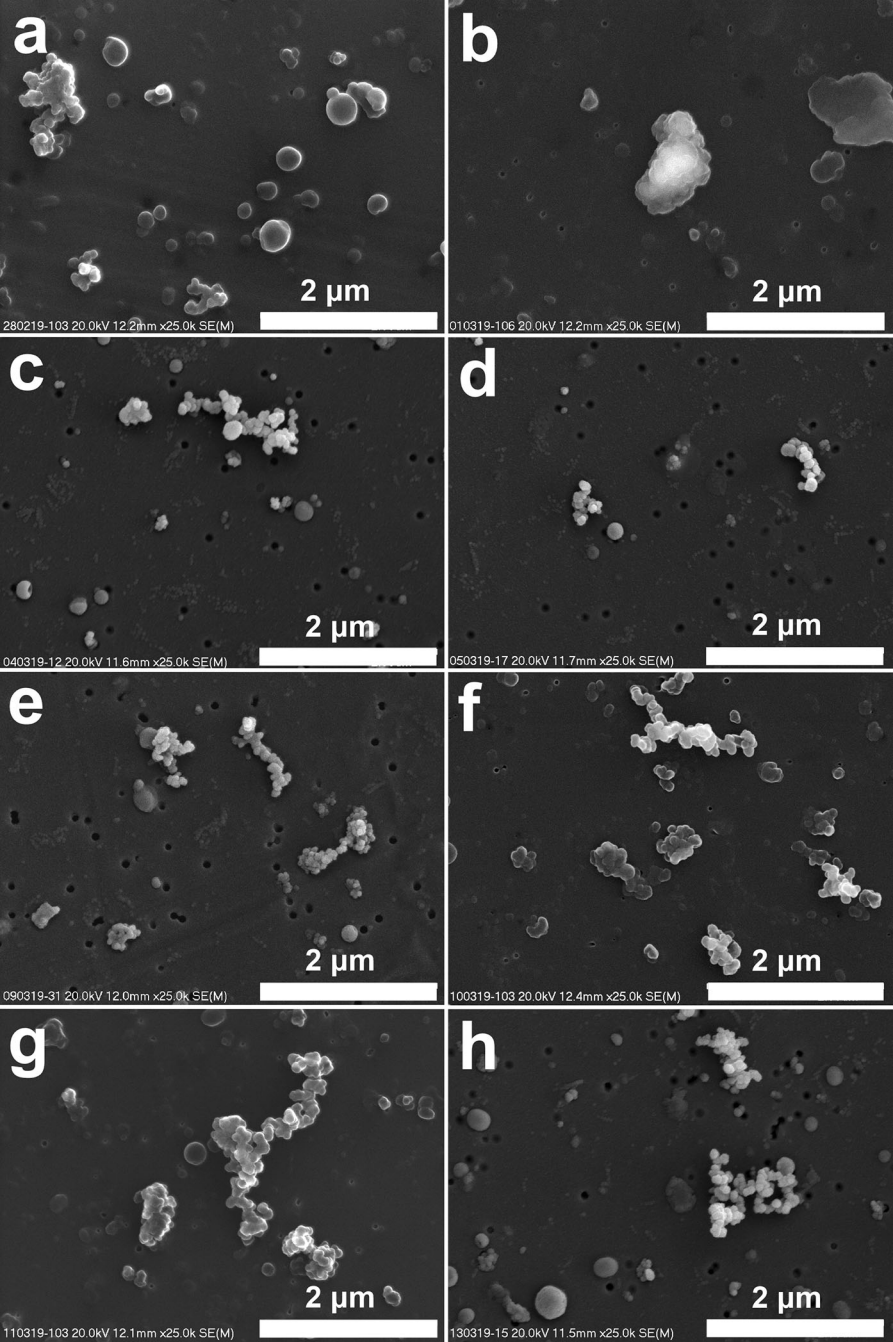
Atmospheric particulate matter on filter (SEM images, magnification 25 000 x). Soot and tar ball-type particles below 1 μm. Images represent different samples: **a**—28/02/2019; **b**—01/03/2019; **c**—04/03/2019; **d**—05/03/2019; **e**—09/03/2019; **f**—10/03/2019; **g**—11/03/2019; **h**—13/03/2019

**Fig. 5:**
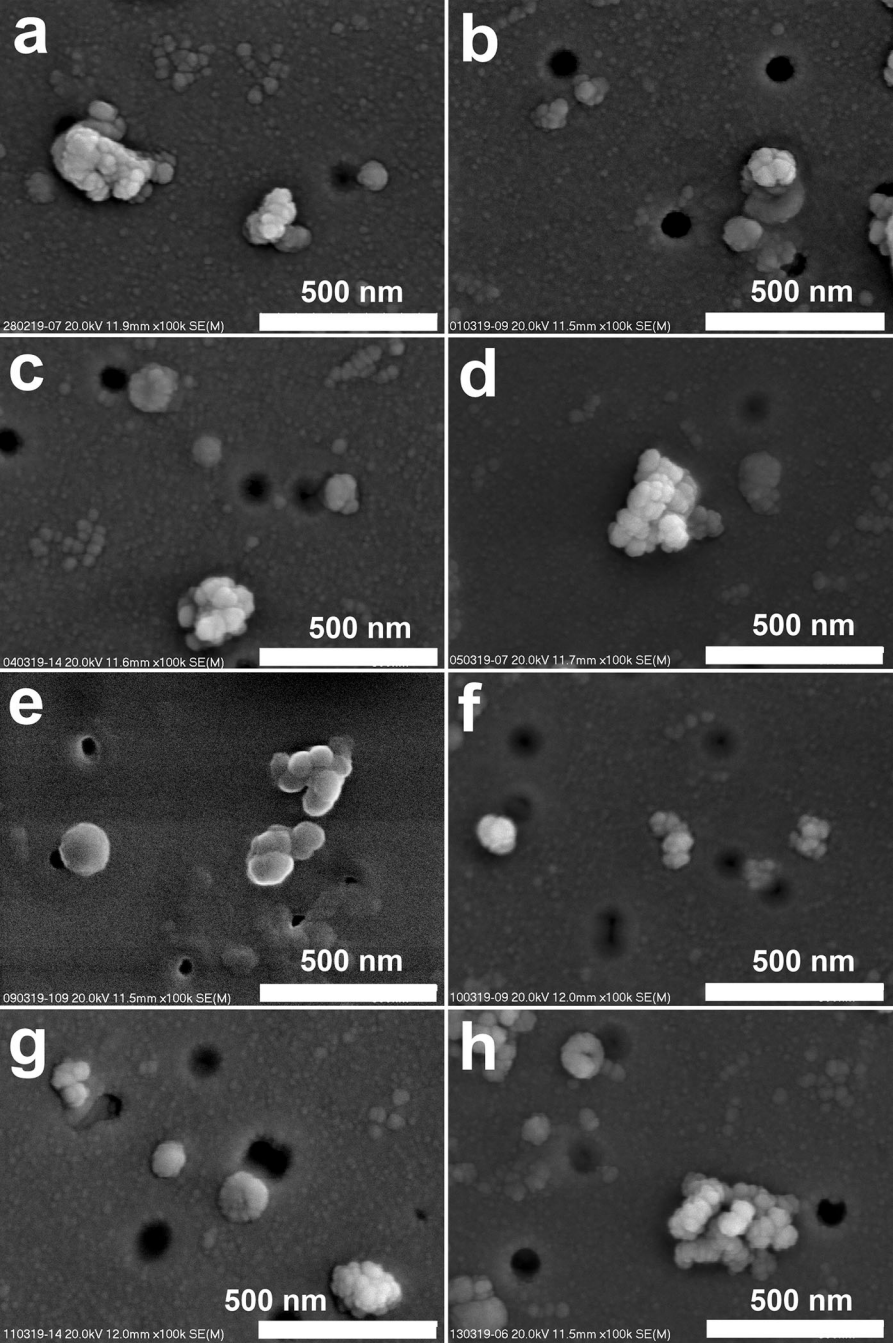
Particles on filters (SEM images, magnification 100 000 x). Soot and single tar ball particle (e) around and below 100 nm. Images represent different samples: **a**—28/02/2019; **b**—01/03/2019; **c**—04/03/2019; **d**—05/03/2019; **e**—09/03/2019; **f**—10/03/2019; **g**—11/03/2019; **h**—13/03/2019

A dominant number of particles in all the studied samples are sized below 1 μm (Fig. 3 and 4). The dominance of PM1 in the PM in Krakow was pointed out by Wilczyńska-Michalik et al. (2015b). The number of particles below 0.1 μm (ultrafine particles) is very high in the studied samples (Fig. 5). Ultrafine particles (below 0.1 μm or 100 nm) are often reported as nanoparticles, but in the case of aerosols, 50 nm is also considered as a boundary of nanoparticles. Kumar et al. (2010) suggested using the < 300 nm boundary for atmospheric nanoparticles, because this size range includes more than 99% of the total particle number concentration in ambient atmosphere. Scanning electron microscope (SEM) observations indicated that a similar particle size distribution is noted in Krakow.

The content of PM1 or PM0.1 in Krakow is not measured systematically. Concentration of PM2.5 is measured at three stations from the State Environmental Monitoring system. Considering the average concentration of PM2.5 and PM10 measured in Krakow in cold (1 November–31 March; average month temperature is below 0 °C) and warm (1 April–31 October; average month temperature is above 0 °C) periods, it is possible to calculate that PM2.5 fraction constitutes 72.88–76.22% in the cold season and 56.11–67.03% of PM10 in the warm season during the 2008–2019 period. There are only limited data related to the particle number concentration in the atmosphere in Krakow. According to Bogacki et al. (2010), the number of particles per 1 m^3^ varied from negligible to more than 400 000 in relation to temperature within a 24-h period.

The common opinion is that the percentage of fine and ultrafine particles in air pollution is growing and will continue to increase along with changing emission sources. The results of the modeling presented by Keogh et al. (2014) indicated that it is possible to expect a 100-fold growth of the particle number concentration in the atmosphere, together with a 31–36% decrease of mass concentration in Southeast Queensland, Australia, up to 2026.

The concentration of nanoparticles in the urban atmosphere (both natural and anthropogenic) is higher than in other environments (Buseck and Adachi 2008). Anthropogenic nanoparticles in the urban environment originate from different sources with an important share of particles from vehicular engines (Kumar et al. 2010, 2011a; Morawska et al. 2009). Nanoparticles in the atmosphere could be primary and secondary. In the urban environments, products of fuel combustion in vehicle engines are the main source of secondary nanoparticles (Morawska et al. 2009). Kumar et al. (2011b) discussed the importance of the measurement of the number and the number–size distribution of nanoparticles in the urban atmosphere and the technical problems related to the regulations and standardization of methods for monitoring. The lung deposited surface area (LDSA) of PM concentration, or the lung deposited surface area size distribution, is considered as a relevant metric for the negative health effects of aerosol particles (Kuuluvainen et al. 2016).

Outdoor PM2.5 is considered as the fifth leading risk factor for death in the world (Schraufnagel et al. 2019). Ultrafine particles cause a greater inflammatory response than fine particles (Donaldson and Stone 2003). The effect is enhanced by the presence of ozone (Oberdörster 2001). Oberdörster et al. (2005) considered nanotoxicology as an emerging discipline because of the great hazard to the biosphere and human health related to exposure to nanoparticles. Nanoparticles are significantly more active biologically than larger particles of the same chemical composition, because of the much greater surface area per mass. The toxicology of inhaled nanoparticles was discussed by Bakand et al. (2012).

#### Carbonaceous products of fuel combustion

Carbonaceous products of fuel combustion dispersed as aerosol particles in the atmosphere influence climate, visibility and health. Their definitions (i.e., tar balls, black carbon and soot) were presented by Buseck et al. (2012). We use the term ‘soot’ for carbon spherical particles below 100 nm in size, which are often aggregated in chain-like or other forms, and tar ball-type particles for larger, single spherical carbon-dominated particles around 200–1000 nm in size.

##### Tar ball-type particles

**Fig. 6:**
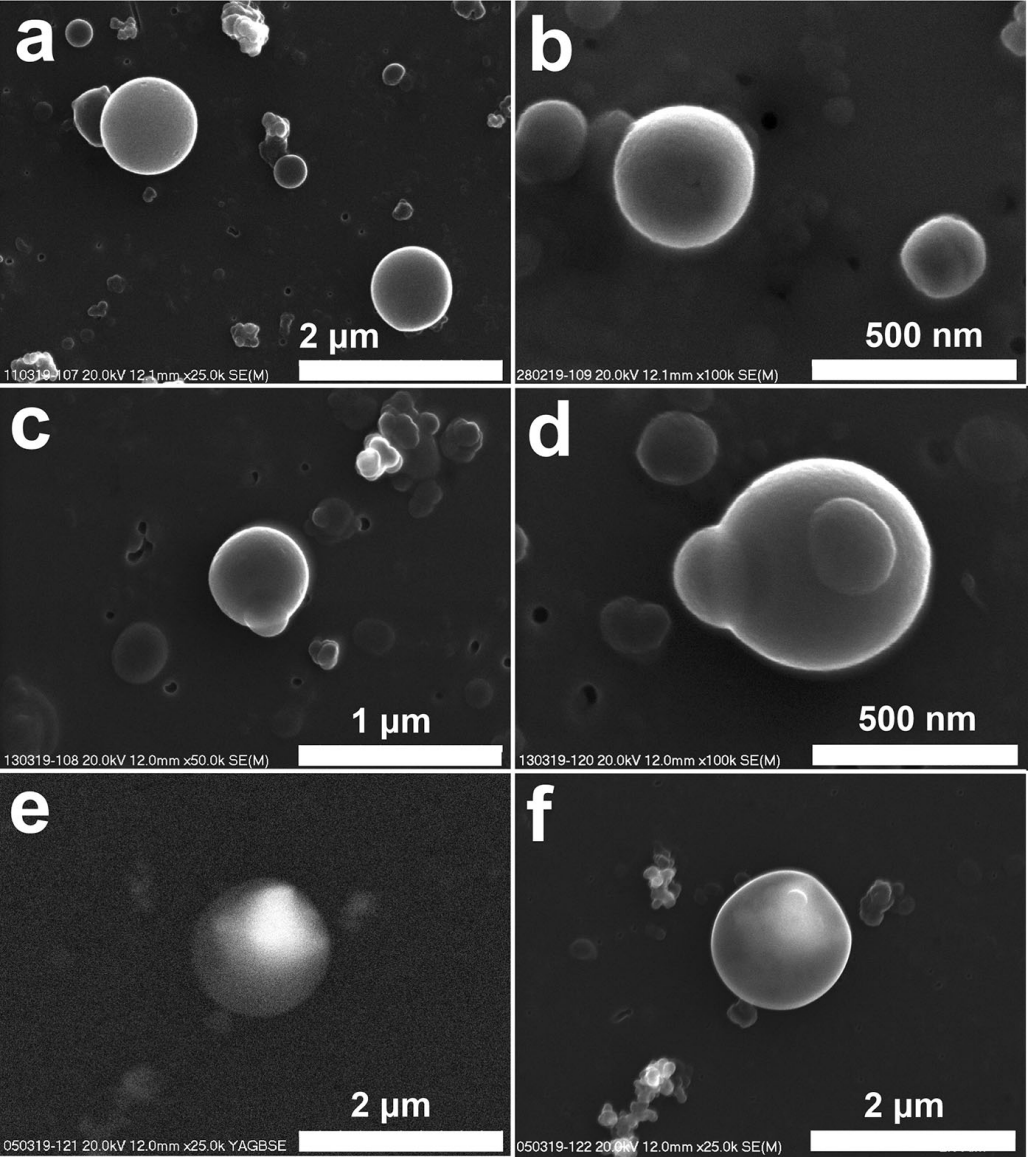
Tar ball-type particles of different size and morphology (SEM, a to d—secondary electrons images). **a** to **c**. Tar balls of the size from 200 nm to 1 μm (**a**—11/03/2019; **b**—28/02/2019; **c**—13/03/2019), **c** and **d**. Tar ball-type particles—smaller hemispherical forms joined to larger spheres (**d**—13/03/2019), **e** and **f**. Tar ball-type particle with elevated content of Ba and S (**e**—backscattered electrons image; **f**—secondary electrons image). Brighter zones (**e**) are enriched in Ba and S. (**e**, **f**—05/03/2019)

Tar ball-type particles in aerosols in Krakow have not been described in detail previously. Their abundant presence was noted in all samples collected in February and March 2019 (Fig. 6). Usually, tar balls are interpreted as a product of biomass burning (Posfai et al. 2004; Adachi and Buseck 2011; China et al. 2013; Adachi et al. 2019). In general, it is assumed that the relative abundance of tar balls increases with the age of the smoke (Adachi and Buseck 2011). Zhang et al. (2018) and Makonese et al. (2019) described the formation of spherical organic particles during residential coal combustion. Makonese et al. (2019) noticed that the size of the spherical particles formed during coal combustion varied within a broad range up to ‘giant’ ones (larger than 2 μm). Tar balls were described as a component of the urban aerosol in the winter period in China (Hu et al. 2012). Taking into account the number of particles in PM2.5, tar balls constitute 9.0–12.9%, where a higher concentration was noted during cloudy days (Hu et al. 2012). Tar balls collected in Krakow were not analyzed using a transmission electron microscope, and we could not prove that they are amorphous (cf. Tóth et al. 2014). As mentioned, the size of the tar balls varies within a broad range from around 200 nm to 1 μm (Fig. 6a–c). This indicates that the typical size of tar balls noted in aerosols in Krakow is larger than described in the literature, both for natural and experimentally produced forms from biomass combustion, while the size can be partially related to the type of biomass burned (Adachi and Buseck 2011; Pósfai et al. 2004; Tóth et al. 2014).

The presence of large tar ball particles can indicate their origin in the coal combustion process. Tar balls usually occur as single spheres, but sometimes smaller hemispherical forms are joined to larger spheres (Fig. 6c, d). The chemical composition of the analyzed tar balls is dominated by C, with low content of O, which can be considered as typical for tar balls (Pósfai et al. 2004; Tóth et al. 2014). In the analyzed tar balls, low content of S, K and Si is common, which was often noted (e.g., Toth et al. 2014). In several tar balls, Ba and S were noted (Fig. 6e–f), and in one tar ball, there was a low content of Zn. According to Zhang et al. (2018), spherical organic matter particles formed during coal combustion contained more Si than tar balls from biomass combustion. Ba and S occur often in products of coal burning (e.g., Wilczyńska-Michalik et al. 2019), but are also present in ash from biomass burning (e.g., Wilczyńska-Michalik et al. 2018). Therefore, it seems that the content of Ba and S in tar balls cannot be considered as an indicator of their source.

The small size and composition of tar balls (dominance of aromatic compounds; Li et al. 2019) indicate their potential hazardous impact on human health. Negative health impact of polycyclic aromatic hydrocarbons from incomplete combustion related to their carcinogenic toxicity is discussed by numerous authors (e.g., Shrivastava et al. 2017).

##### Soot particles

Soot is a common component of atmospheric pollution that intensively absorbs solar and terrestrial radiation, the second factor after CO_2_ that causes global warming (Wang et al. 2016).

**Fig. 7:**
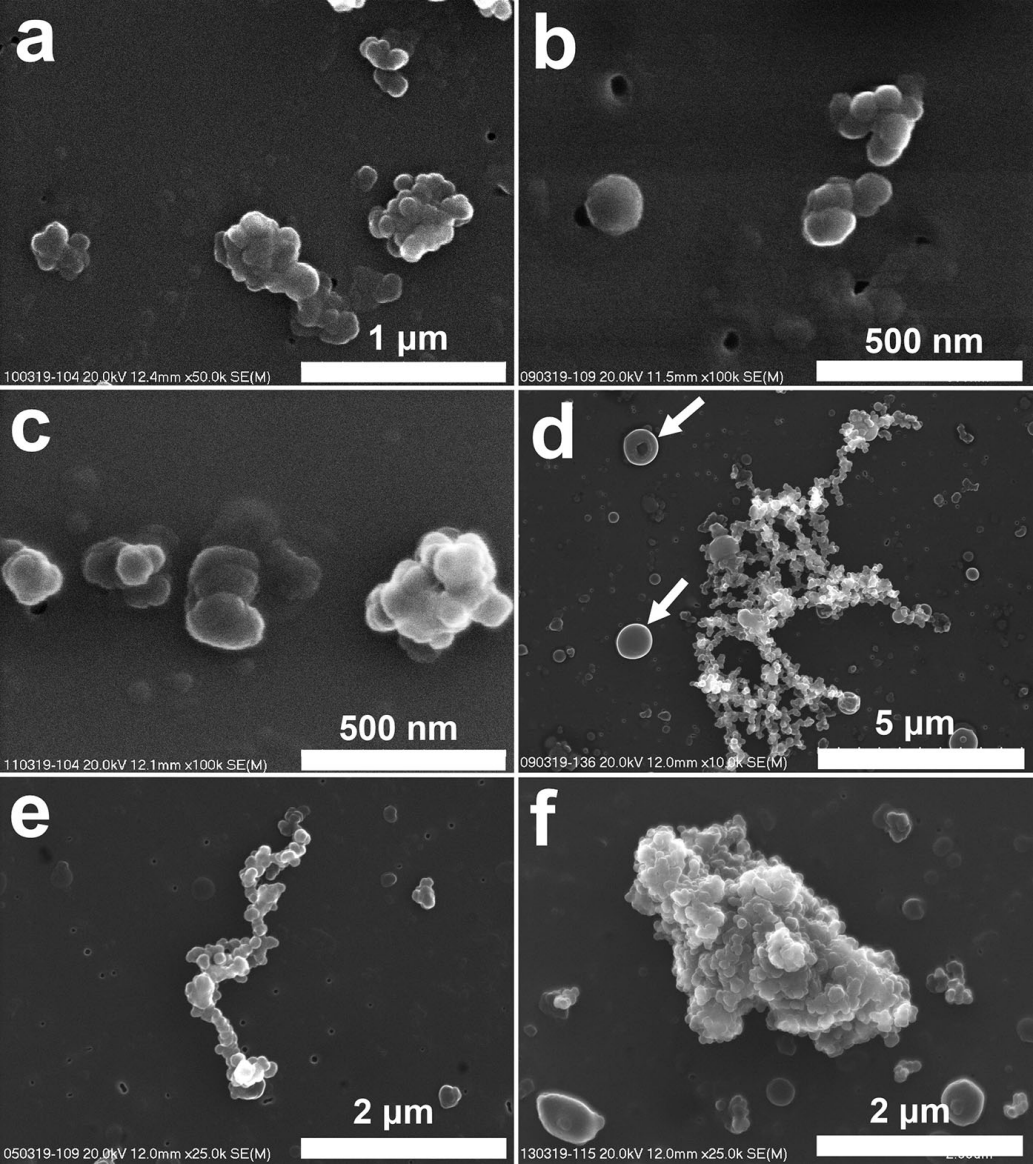
Soot particles (SEM, secondary electron images). **a** to **c**. Soot—small agglomerates composed of several particles and single particles (**a—**10/03/2019; **b**—09/03/2019; **c**—09/03/2019), **d**. Loosely packed agglomerate of numerous soot particles; tar balls (arrows) (09/03/2019), **e**. Lacey-type agglomerate of soot particles (05/03/2019), **f**. Compact agglomerate of soot particles (13/03/2019)

Soot is a common component of aerosol particles collected in Krakow. It can be present as single particles (around 100 nm in size or smaller) (Fig. 7a, b), although agglomerates occur more often (Fig. 7b, c, d). These agglomerates could be small, composed of several particles (Fig. 7b, c), or larger, containing numerous particles (Fig. 7d). Soot occurs as lacey and compact agglomerates (Fig. 7e, f). Loosely packed agglomerates reach 20 μm. The formation of compact forms is related to aging, due to condensation and evaporation of water on their surface (e.g., Zuberi et al. 2005). Soot identification in the studied samples was based on the morphology of particles or agglomerates (and confirmed by C-dominated chemical composition). The morphology of soot (particle size and the shape of agglomerates) cannot be used for source identification (Michalik et al. 2016).

The health hazards of soot (e.g., high carcinogenic, mutagenic and allergenic potential) are discussed in numerous papers (e.g., Niranjan and Thakur 2017; Saenen et al. 2017; Shiraiwa et al. 2012; Su et al. 2008). Soot originates from incomplete combustion of fuels, and it contains various amounts of volatile or semi-volatile organic substances (including polycyclic aromatic compounds, alkenes and carboxylic acids) and metals (Kelly and Fussell 2015; Niranjan and Thakur 2017; Shiraiwa et al. 2012). The characteristics of soot (i.e., the content of organic substances) are related to the type of fuel and combustion conditions (Atiku et al. 2016). Su et al. (2008) determined that the cytotoxic and inflammatory potential of soot from Euro IV is higher than soot from old diesel engines, mostly due to the smaller particle size (the high abundance of chemically reactive edges and the presence of surface functional groups). Also, Stone et al. (1998) concluded that ultrafine black carbon (soot) exhibits a stronger effect on health than fine black carbon. Soot particles, because of their small size, can penetrate deeply into the human body; for example, Bové et al. (2019) described the presence of soot in human placenta, while Saenen et al. (2017) recognized airborne carbon (soot) particles in the urine of children, which indicates the translocation of these ultrafine particles from the lung to the circulation and then to the urine.

The content of metals in individual soot particles is rather low, but taking into account the number of particles, the health impact could be significant. Using the SEM–EDS method, it was possible to measure the Si, Na and S content in some particles. It is possible to thus assume that the cumulative effect of soot and other particles can be important. The synergistic adverse health effects of soot and Fe-rich particles were described by Zhou et al. (2003).

#### Metals in PM

Metals contained in atmospheric PM exert various health effects (Cakmak et al. 2014; Chapman et al. 1997; Fortoul et al. 2015; Gonet and Maher 2019; Maher 2019). The content of metals in atmospheric PM was analyzed by numerous authors using different methods. Metal-containing particles differ significantly in chemical and physical characteristics (Sanderson et al. 2014). Rogula-Kozłowska et al. (2014) compared the mass concentration and chemical composition of PM2.5 samples collected in urban sites (Katowice and Gdańsk) and a regional background site (Diabla Góra) in Poland. Pastuszka et al. (2010), Rogula-Kozłowska and Klejnowski (2013), Rogula-Kozłowska et al. (2015) and Samek et al. (2017) presented results of analyses (including the chemical composition) of PM from different urban sites in southern Poland. Krzemińska-Flowers et al. (2006) measured the concentration of trace elements in PM in three sites in the Polish city of Łódź.

The concentration of four metals considered to be toxic (Pb, As, Cd, Ni) is measured in PM10 within the frame of the European monitoring system. Data from European countries in 2017 indicated that the target value for As was exceeded in seven (three in Poland) out of 645 stations, for Cd in two stations out of 670 and for Ni in five stations out of 649, while Pb was below the target value in all the data from 642 stations (Air Quality in Europe—2019 report).

Analyses of metals contained in single particles of atmospheric dust in Poland are relatively scarce. Wawroś et al. (2003) determined particles rich in Fe, Cu, Ti, Cr, Zn and Ni in samples collected in Katowice, while Wilczyńska-Michalik and Michalik (2015) and Wilczyńska-Michalik et al. (2015a, b) described particles rich in metals in PM from Krakow.

The details of the single-particle analysis of aerosol samples collected in February and March 2019 in Krakow indicate that both the content and characteristics of metal-rich particles in all samples are similar. However, the number of analyzed particles was too low for statistically valid determination of the content of different groups of particles. Particles containing different metals, except for alkaline and alkaline-earth metals (but including Ba), and some metalloids will be discussed here.

**Fig. 8:**
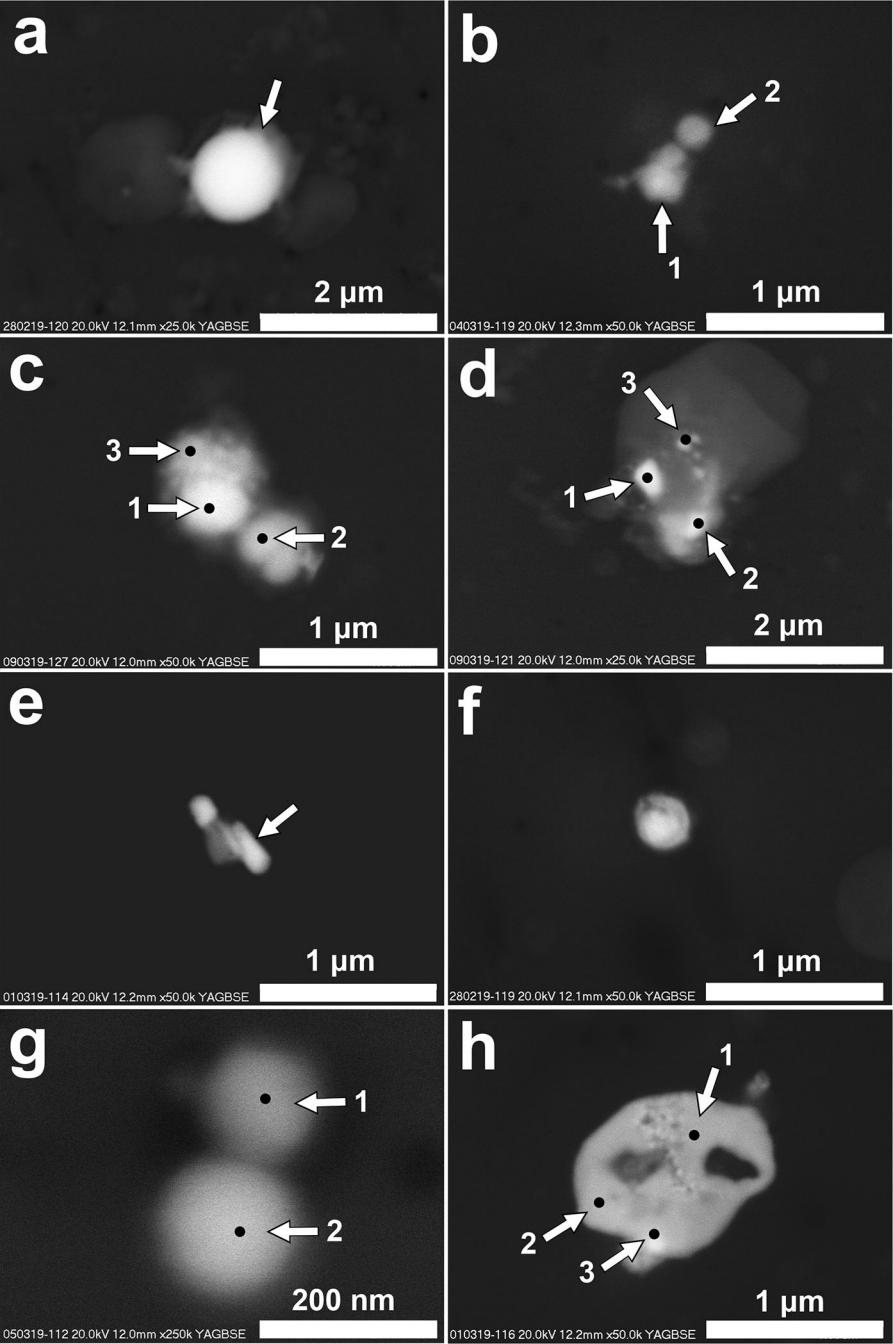
Metal-rich particles (SEM, backscattered electrons images; content of major components presented for selected analytical spots). **a**. Spherical Fe-rich particle (arrow) (Fe—52.5wt%; Mn—23.1wt%; Zn—1.2wt%; O—17.3wt%); **b**. Particles rich in Mn (particle 1: Mn—61.1wt%; Zn—2.9wt%; Fe—8,4wt%; O—21.7wt%; Si—5.99wt%; particle 2: Mn—66.1wt%; Zn—2.1wt%; O—22.4wt%; Si—9.5wt%); **c**. Particles rich in metals (spot 1: Fe—32.5wt%; Mn—5.0wt%; Zn—20.3wt%; Cr—4.1wt%; Ni—1.8wt%; Mg—9.6wt% O—18.0wt%; spot 2: Fe—34.7wt%; Mn—4.3wt%; Zn –21.5wt%; Cr—2.7wt%; Ni—1.4wt%; Mg—7.8wt%; O—18.8wt%; spot 3: Fe—22.7wt%; Mn—4.0wt%; Zn—9.2wt%; Cr—25.9wt%; Ni—1.2wt%; Mg—8.2wt%; O—21.3wt%); **d**. Cu-rich particles on aluminosilicate grain (spot 1: Cu—87.owt%; O—10.2wt%; spot 2: Cu 94.4wt%; O—3.9wt%; spot 3: Cu—86.6wt%; O—11.7wt%); **e**. Particle rich in Pb (Pb—60.1wt%; O—28.2wt%; Fe—2.7wt%; S—9.0wt%); **f**. Particle rich in Sn and Pb (Sn—53.7wt%; Pb—11.2wt%; O—35.1wt%); **g**. Small spherical Fe-rich particles (spot 1: Fe—65.7; Mn—6.33wt%; Zn—3.5wt%; Cr—1.1wt%; O—22.5wt%; spot 2: Fe—74.9wt%; Mn—2.8wt%; O—20.7wt%); **h**. Particle rich in Pb, Cl and O (spot 1: Pb—52.6wt%; O—18.6wt%; Cl—26.8wt%; spot 2: 52.6wt%; O—20.1wt%; Cl—24.7wt%; spot 3; Pb—49.3wt%; O—19.0wt%; Cl—12.1wt%; S—8.9wt%; K—9.7wt%)

The most common group is dominated by a relatively high content of metals and oxygen. Generally, it is difficult to precisely determine the content of oxygen in metal-rich particles using the EDS method, but most of the particles in this group can be considered to be oxides or oxide-dominated. Usually, Fe is a dominant metal with the content above 50 wt%. Fe is often accompanied by Mn and Zn in various proportions (Fig. 8a). The content of Mn varies from 0 to more than 60 wt%. In particles with Mn content higher than 20 wt%, Mn usually predominates over Fe. One particle with 66.1 wt% of Mn is devoid of Fe, but contains ~ 2 wt% of Zn (Fig. 8b). In several particles, high content of Zn was measured (**≥ **20 wt%). Cr often co-occurs in Fe-rich particles containing Mn or Zn (Fig. 8c). Usually, the content of Cr is relatively low, but sporadically it is higher (e.g. >25 wt%) and dominates over Fe. Cu co-occurs with other metals in Fe-rich particles, but it is also noted as only one admixture in Fe-rich particles. In several particles, Cu was found as only one metal and its content was above 85 wt% (Fig. 8d). Ni occurs rarely, usually in Fe-rich particles containing Mn, Zn and Cr. Particles rich in Pb (> 60 wt%) and O (with low content of Fe and S) were noted (Fig. 8e), as well as particles containing Sn (>50 wt%), Pb (>11 wt%) and O (>35 wt%) (Fig. 8f). Low content of Sn was determined in Fe-rich particles also containing Mn, Zn and Cr. Low content of Sb (slightly above 1.5 wt%) was noted in Fe-rich particles containing low content of Cu. In several Fe-rich particles, Ba was noted. In numerous particles, Si, Al, Ca, Mg, Na, S, Cl and K were noted. Particles with very high content of Fe (ca. 90 wt% or higher) or Cu (>90 wt%) are characterized by very low content of O. It is possible to consider them as slightly oxidized metallic particles.

Fe-rich particles (oxides) commonly occur as irregular forms varying in size from several micrometers to tens of nanometers. Spherical Fe-rich particles are less common than irregular ones. The size of spherical Fe-rich particles varies from several micrometers to below 100 nm (Fig. 8g). Small spherical Fe-rich particles (ca. 100 nm) often occur in clusters containing several particles slightly differing in size (above and below 100 nm). The occurrence of Fe-rich spherical particles around or below 100 nm in diameter, according to our knowledge, has not been noted in atmospheric PM in Krakow. The study by Moteki et al. (2017) indicated the presence of aggregates of numerous anthropogenic FeOx monomers with sizes ranging from several to 100 nm in aerosol from the East Asia. Similar aggregates of spherical Fe oxides were described from the atmosphere of megacities (e.g., Tokyo; Adachi et al. 2016). According to Sanderson et al. (2016), Fe nanoparticles occurring in the atmosphere of urban environments can be related to road traffic (formed in engine cylinders or braking systems). Liati et al. (2015) found that road traffic metal nanoparticles are derived mainly from metal fragments melting in diesel engines. Xing et al. (2019) noticed Fe nanoparticles in material emitted from gasoline direct-injection engines. The origin of particles of this type from metallurgical industrial sources is probable (Jenkins 2003). The small spherical Fe-rich particles are similar to the airborne particles detected in the human brain by Maher et al. (2016). The health impact of Fe-bearing nanoparticles in urban environments was discussed by Gonet and Maher (2019).

Low S content was noted in numerous particles, but higher concentrations corresponding to values typical for sulfates were rarely noted. Ca sulfates (sometimes containing low amounts of Cl, Na, K, Si, Al) were noted. The complex chemical composition indicates the internally mixed state of these particles. Ba sulfate (barite) particles were also found. Single particles of Fe- or Zn-rich sulfates were also noted.

Cl-rich particles containing metals were rarely determined. All of them were characterized by high Pb content (ca. 50 wt%). The content of O indicates that the particles are not simple chlorides (Fig. 8h). The presence of S noted in one particle, and K, Si and Al in others, suggests their chemical complexity.

Determination of the carbonates in aerosols using the SEM–EDS method and carbon-coated samples is doubtful. Particles rich in Ca and Mg characterized by very low content of Si and Al were considered to be carbonates. The content of transition metals in these particles is low (e.g., Fe rarely above 3 wt%; Mn up to 1 wt%).

Relatively numerous particles containing transition metals and relatively rich in Si and Al were arbitrary included into the group of aluminosilicates. The content of Fe varies from 2 to 39 wt%. In some particles, Mn is present (0.5–32.9 wt%), along with Zn (1–2 wt%), Ti (0.3–20.6 wt%) and Ba (ca. 1 wt%). The aluminosilicates often contain low amounts of K, Ca, Mg, P, S and Cl. The majority of the aluminosilicate particles are irregular, with spherical ones occurring less often. These spherical particles are usually below 2.5 μm in size, while some are around 100 nm.

There is only a scarce occurrence of particles (< 2.5 μm in size) composed of Zr (32–62 wt%), Ce (8–16 wt%), O and Al, likely originating from catalyst material used in diesel engine vehicles (Davies et al. 2018).

Single-particle analysis of various types of metal-bearing airborne particles in the studied samples cannot be considered as an indicator of the concentration of metals in aerosols or as a measure of their size segregation. Variation in the forms of occurrence of metals in aerosol particles, as well as in the size of particles, indicates the variability of their properties, but determination of these properties (e.g., chemical speciation, oxidation state, solubility and bioavailability) requires more advanced studies. Generally, the bioavailability of metals increases with decreasing PM size (Chapman et al. 1997).

The abundance of particles containing transition metals (with high content of metal oxides) in the studied samples indicates their potential for adverse health impacts through oxidative stress (Biswas and Wu 2005; Gonet and Maher 2019; Kelly 2003; Manke et al. 2013; Sørensen et al. 2005). Oxidative stress is formed according to Fenton and Haber–Weiss reactions (Biswas and Wu 2005; Kanti Das et al. 2015). Fe nanoparticles are important in the development of oxidative stress (Biswas and Wu 2005; Gonet and Maher 2019). The redox activity of metals in PM depends not only on the content of metals, but also on the bioavailability, and the content of other components such as sulfates (Nawrot et al. 2009). Gaseous organic compounds can increase metal solubility via metal–organic complexation (Okochi and Brimblecombe 2002).

### Microbiological analysis

Twelve different species of bacteria were isolated from the collected aerosol samples. The most frequently isolated species was Gram-positive sporulating *Bacillus licheniformis* (isolated four times). This was a similar result to the one obtained by Brągoszewska and Pastuszka (2018), who also found that the most prevalent bacteria in outdoor air in Gliwice were Gram-positive rods forming endospores, while *Bacillus cereus* was the most commonly isolated bacterium, and *B. licheniformis* was not found. In our study, seven other *Bacillus* species were identified in various samples, while in the aforementioned study by Brągoszewska and Pastuszka (2018), only four different species were found. Contrary to another study by Brągoszewska et al. (2020), concerning indoor and outdoor microbiological air quality in a high school gym in Southern Poland, we did not isolate *Corynebacterium*. The reasons for such results are probably the differences in air sampling and the identification method. Brągoszewska et al. (2020) used an Andersen six-stage impactor ANDI for sampling, but in case of identification, the MALDI-TOF method was used in our study, while biochemical API tests were utilized in the cited studies. Among the staphylococci isolated from the aerosol samples collected from air, there were two different species: *Staphylococcus warneri* and *Staphylococcus epidermidis*. Among the streptococci isolates, there were *Streptococcus salivarius*, *Streptococcus parasanguinis*, *Streptococcus vestibularis* and *Streptococcus sanguinis*. Although there was no medium dedicated to finding fungi, some colonies of to these organisms were demonstrated through the use of blood agar. Identification tests were also performed in this case. The isolated mold fungi belonged to the genus *Aspergillus*. In most cases, microbial growth was obtained both by direct culture on agar and after pre-incubation on tryptic soy broth. Only in two samples, where the culture on agar was negative, bacterial growth was obtained after pre-incubation in the broth. In quantitative cultures, the total number from 2 * 10^2^ CFU / ml to 5 * 10^8^ CFU / ml of bacteria was found. Detailed results of the microbiological testing are presented in Table 3.

**Table 3:** Results of microbiological analysis of aerosol samples

Date (dd/mm/yyyy)	Microorganism morphology (result of Gram stained)	Species (qualitative culture)	Species (quantitative culture)	CFU/ml tryptic soy agar	CFU/ml Columbia agar
28/02/2019	Gram-positive sporulating bacilli	*Bacillus licheniformis*	*Bacillus licheniformis*	6 × 10^7^	4 × 10^7^
	Gram-positive sporulating bacilli	*Paenibacillus barengoltzii*	*Paenibacillus barengoltzii*	1.6 × 10^6^	1.1 × 10^8^
	Gram-positive cocci	*Staphylococcus warneri*	*Staphylococcus warneri*	–	6 × 10^6^
	Gram-positive cocci	*Rothia terrae*	–	–	–
	Gram-positive cocci	*Micrococcus luteus*	–	–	–
	Molds	–	*Aspergillus sp.*	single	–
1/03/2019	Gram-positive sporulating bacilli	*Bacillus licheniformis*	*Bacillus licheniformis*	3 × 10^8^	1 × 10^8^
	Gram-positive sporulating bacilli	*Bacillus pumilus*	*Bacillus pumilus*	1.6 × 10^6^	3 × 10^6^
	Gram-positive cocci	*Staphylococcus epidermidis*	–	–	–
	Gram-positive sporulating bacilli	–	*Bacillus altitudinis*	4 × 10^7^	–
4/03/2019	Gram-positive sporulating bacilli	Bacillus simplex	*Bacillus simplex*	2 × 10^6^	1 × 10^6^
	Gram-positive sporulating bacilli	Bacillus clausii	*Bacillus clausii*	3 × 10^6^	–
	Gram-positive cocci	Streptococcus salivarius	*Streptococcus salivarius*	–	9 × 10^5^
	Gram-positive cocci	Streptococcus parasanguinis	*Streptococcus parasanguinis*	–	2 × 10^5^
	Gram-positive sporulating bacilli	–	*Brevibacillus borstelensis*	3 × 10^6^	–
5/03/2019	Gram-positive sporulating bacilli	*Bacillus licheniformis*	*Bacillus licheniformis*	5 × 10^8^	1 × 10^8^
	Gram-positive cocci	*Streptococcus vestibularis*	*Streptococcus vestibularis*	–	1.6 × 10^7^
9/03/2019	Gram-positive sporulating bacilli	*Bacillus amyloliquefaciens*	*Bacillus amyloliquefaciens*	4 × 10^8^	1 × 10^7^
	Gram-positive sporulating bacilli	*Bacillus cereus*	*Bacillus cereus*	2 × 10^7^	3 × 10^8^
	Gram-positive sporulating bacilli	*Bacillus pumilus*	*Bacillus pumilus*	1.3 × 10^6^	2 × 10^6^
	Gram-positive cocci	*Streptococcus sanguinis*	*–*	–	–
10/03/2019	Gram-positive non-sporulating bacilli	*Lactobacillus kefiri*	*Lactobacillus kefiri*	5 × 10^2^	3 × 10^1^
	Gram-positive sporulating bacilli	*Bacillus subtilis*	*Bacillus subtilis*	2 × 10^2^	1.5 × 10^2^
	Gram-negative rods	*Pantoea agglomerams*	–	–	–
	Molds	*Aspergillus niger*	–	–	–
11/03/2019	Gram-positive sporulating bacilli	*Bacillus cereus*	*Bacillus cereus*	1.7 × 10^8^	2 × 10^8^
	Gram-positive sporulating bacilli	*Bacillus altitudinis*	*Bacillus altitudinis*	1 × 10^7^	4.2 × 10^7^
	Molds	*Aspergillus fumigatus*	–	–	–
	Gram-positive cocci	*Staphylococcus warneri*	*Staphylococcus warneri*	–	1 × 10^6^
	Gram-positive non-sporulating bacilli	*Lactobacillus plantarum*	–	–	–
	Gram dodatni ziarenkowiec	*Micrococcus luteus*	–	–	–
	Gram-negative rods	*Stenotrophomonas maltophilia*	–	–	–
13/03/2019	Gram-positive sporulating bacilli	*Bacillus altitudinis*	*Bacillus altitudinis*	1.4 × 10^7^	9 × 10^6^
	Gram-positive sporulating bacilli	*Bacillus licheniformis*	*Bacillus licheniformis*	8 × 10^7^	1 × 10^7^
	Gram-positive non-sporulating bacilli	*Lactobacillus plantarum*	–	–	–
	Gram-positive cocci	*Micrococcus luteus*	–	–	–

The most common bacteria found during the study that can cause infections in humans are *B.cereus*, *Stenotrophomonas maltophilia*, *S. warneri* and *S. epidermidis.*

*Bacillus cereus* is an opportunistic pathogen causing primarily food intoxication but also other infectious diseases. Besides soil, which is its primary reservoir, it can be isolated from vegetation and water and can colonize mammals. From the environment, Bacillus spores can be transferred into various raw materials used in the food industry. The host can be contaminated by spores or vegetative cells present in food, inhaled air, or enter the body through a wound (Ehling-Schulz et al. 2019, Ramarao et al. 2020). *Stenotrophomonas maltophilia* is a commensal and a pathogen that is more and more often isolated from people with normal immunity. This bacterium is also more and more frequently resistant to antibiotics. It is involved in skin and soft tissue infections, bloodstream infections, endocarditis, meningitis, acute respiratory infection and others. *S. maltophilia* is an environmental bacterium found in water and as part of the microflora of animals. The route of transmission is the contact route through both healthy and injured skin and mucous membranes (Adegoke et al. 2017). *S.warneri* and *S. epidermidis* are coagulase-negative staphylococci, which are part of the physiological microbiota of the skin and mucous membranes. They are also important pathogens, especially in hospitals, because it is more common for them to infect immunocompromised patients. The main route of transmission for *Staphylococcus* bacteria is the contact route, but indoor air testing indicates the presence and the possibility of long-term survival of numerous bacteria of this genus in the air (Becker et al. 2014; Lee et al. 2019; Lenart-Boroń et al. 2017).

In patients with severely compromised immune systems, any organism may be potentially pathogenic. *Aspergillus* species are also dangerous pathogens, primarily for people with specific predisposing factors. Tham et al. (2017) found that exposure to the spores of several outdoor fungal taxa, including *Alternaria*, *Leptosphaeria*, *Coprinus* and *Drechslera* species, was associated with the risk of asthma exacerbations in children and adolescents, regardless of their sensitization to *Alternaria* and *Cladosporium* species. In our study, these species were not found. Our results concerning the fungal composition of bioaerosols are contrary to the results of Bugajny et al. (2005), where in the outdoor air in Poznań, they found higher concentrations of fungi than bacteria, and with more diverse composition (*Cladosporium spp*., *Mucor spp*. and *Alternaria spp*.). The difference probably results from the fact of choosing a medium dedicated to bacterial culture in our study.

The results of microbiological analysis should be treated as preliminary ones, due to limitations in sampling as well as the selected method of microorganism identification. Our method for microbiological tests did not allow to identify the viable but not culturable bacteria and a broad diversity of fungi or viruses. According to Ravva et al. (2012) and Fahlgren et al. (2010), culturable bacteria represent only 1 to 20% of total bacterial diversity; therefore, the method used limited our ability to study the ecology of entire bacterial communities. More recently, increasing accessibility to high-throughput next-generation sequencing (NGS) technologies has enabled identification of hundreds of species and their antimicrobial resistant genes in single metagenomic samples. Metagenomic high-throughput sequencing is currently being explored for expanded use in public health surveillance (Otto, 2017). We believe that our research is only a small introduction and should be continued at the level of both classical and molecular methods.

Additionally, the study was conducted over eight different days during one season of the year, and the diversity of microorganisms between individual days was relatively large. More samples taken in different weather conditions and with several repetitions should thus be taken and subjected to microbiological testing to obtain complex data of microbiological air contamination (and potential health risk), and allow statistical analysis.

More advanced methods of sampling (e.g., Kim et al. 2017), analyses (e.g., Albrecht et al. 2007; Bowers et al. 2009; Gohli et al. 2019) as well as sampling strategy are needed to obtain precise information about the composition of microorganism assemblage and their variations.

## Conclusions

1. The domination of submicron particles in the air pollution in Krakow (and the high concentration of ultrafine particles) indicates that the exposure to ambient air has a very important health impact.
2. Particles originating from fuel combustion dominate in the studied aerosol samples, taking into account the number of particles.
3. Tar balls are relatively common in the studied samples. Their size and composition (aromatic compounds) indicate a potential health impact.
4. Soot occurring in various forms (small or large agglomerates, lacey or compact) is very common in the studied samples. The health impact of soot is strong because of the small particle size, as well as the content of organic compounds and metals.
5. Numerous types of metal-containing particles were identified, differing in the content of the main and accompanying metals and in the chemical form of occurrence. The abundance of transition metals occurring commonly in the form of oxides suggests that these particles could exert a strong and adverse health impact because of strong oxidative stress.
6. The aerosol particles collected in February and March 2019 in Krakow can be considered to indicate an important threat to human health because of the very high content of submicron and ultrafine particles (nanoparticles), the high content of hazardous fuel combustion-related particles (soot and tar ball-type particles) and the abundance of particles rich in transition metals. The detailed characteristics of the particles indicate that the threat is severe, despite the lowering of the mass concentration of PM.
7. The results of this study of bacterial air contamination are generally similar to those of other researchers in Poland conducted with the usage of conventional diagnostics based on culture. Among the isolated and identified bacteria and fungi, some species may cause an infection in vulnerable, immunocompromised patients. The number of isolated species in this study is probably limited due to the method applied.
8. The results of microbiological analysis, which should be treated as preliminary ones, indicate variation in terms of both qualitative and quantitative results. Because of limited number of analyzed samples, it is difficult to interpret this variation.

## Data Availability

(data transparency): Detailed analytical data are stored by the authors and are available on request.

## Code availability

(software application or custom code): Not applicable.
